# Can nasal *Staphylococcus aureus* screening and decolonization prior to elective total joint arthroplasty reduce surgical site and prosthesis-related infections? A systematic review and meta-analysis

**DOI:** 10.1186/s13018-020-01601-0

**Published:** 2020-02-19

**Authors:** Xingyang Zhu, Xiaobo Sun, Yuqing Zeng, Wenjun Feng, Jie Li, Jianchun Zeng, Yirong Zeng

**Affiliations:** 1 The First Clinical Medical School, Guangzhou University of Chinese Medicine, Jichang Road 12#, District Baiyun, Guangzhou, Guangdong China; 2 Yichuan People’s Hospital, Jiuchang Road 21#, District Yichuan, Luoyang, Henan China; 3 Department of Orthopaedics, The First Affiliated Hospital of Guangzhou University of Chinese Medicine, Jichang Road 16#, District Baiyun, Guangzhou, 510405 Guangdong China

**Keywords:** *Staphylococcus aureus*, Surgical site infection, Periprosthetic joint infection, MRSA, Screening, decolonization

## Abstract

**Background:**

Nasal *Staphylococcus aureus* (*S*. *aureus*) screening and decolonization has been widely used to reduce surgical site infections (SSIs) prior to total knee and hip arthroplasty (TKA and THA). However, it remains considerably controversial. The aim of this study was to ascertain whether this scheme could reduce SSIs and periprosthetic joint infections (PJIs) following elective primary total joint arthroplasty (TJA).

**Methods:**

A systematic search was performed in MEDLINE, Embase, and the Cochrane Library until October, 2019. Outcomes of interest included SSI, PJI, superficial infection, and different bacterial species that caused infections. Data from eligible studies were then extracted and synthesized. Pooled odds ratios (OR) and 95% confidence intervals (CIs) were calculated. We also performed additional analyses to evaluate whether there were differences in postoperative SSIs caused by *S*. *aureus* or other bacteria.

**Results:**

Nine studies were included in our meta-analysis. The pooled data elucidated that nasal *S*. *aureus* screening and decolonization dramatically mitigated the risk of SSI, PJI, and superficial infection compared to nondecolonization group. The analysis of bacterial species causing infection also showed that the *S*. *aureus* infections postoperative were significantly decreased in the decolonization group. However, there was no statistical difference in the SSI caused by other bacteria between the two groups.

**Conclusion:**

*S*. *aureus* screening and decolonization prior to elective primary THA and TKA could significantly decrease the risk of SSI and PJI. However, more robust studies are needed to further evaluate the impact of *S*. *aureus* screening and decolonization on infection risk after TJA.

## Introduction

Periprosthetic joint infection (PJI) is a devastating complication following total joint arthroplasty (TJA) and accounts for the majority of postoperative revisions [1, 2]. Meanwhile, it brings a huge economic burden to the healthcare system each year [3–5]. Therefore, the prevention of PJI cannot be overemphasized. Recently, more and more surgeons have emphasized the importance of improving modifiable risk factors preoperatively [6–9]. The nasal *Staphylococcus aureus* colonization is generally considered to be one of the modifiable risk factors [6].

Some studies showed that the routine implementation of nasal screening and selective decolonization could significantly mitigate the risk of surgical site infection (SSI) following TJA [10–13]. However, it is still controversial, because several studies suggested the opposite conclusion [14, 15].

Therefore, the purpose of this systematic review and meta-analysis was to quantitatively evaluate (1) was there any association between the nasal carrying of *S*. *aureus* and the high rate of SSI or PJI after primary elective total hip and total knee arthroplasty (THA and TKA), (2) could preoperative nasal *S*. *aureus* screening and decolonization reduce the rate of SSI and PJI, and (3) was the routine *S*. *aureus* screening necessary before TJA.

## Methods

This study was conducted strictly according to the Preferred Reporting Items for Systematic Reviews and Meta-Analyses statement [16]. Before the start of literature searches, the research protocol was determined by all coauthors.

### Search strategy

Following the PICOS methodology, two authors (Xingyang Zhu and Xiaobo Sun) developed the search strategies with the assistance of an experienced librarian. The last database search was performed on October 18, 2019 with comprehensive strategies, including both Medical Subject Headings and keywords, for the following electronic databases: MEDLINE (PubMed), EMBASE (Elsevier platform), and COCHRANE CENTRAL (through the Cochrane-Library). When searching, we had no restrictions on the language and publication date of the articles to maximize the sensitivity. Additionally, reference lists of relevant articles were also screened to identify additional studies. See Appendix 1 for the full and detailed search strategy**.**

### Inclusion and exclusion criteria

Two independent authors assessed the titles or abstracts or full text of the final search results. In case of any controversy that arose between the two reviewers (Xingyang Zhu and Xiaobo Sun) regarding eligibility, an agreement could be reached through discussion. If no consensus was achieved, the final decision was made by the third author (Yirong Zeng).

The inclusion criteria were listed as follows: (1) all surgical procedures were primary THA or TKA; (2) data detailing SSI rate in patients with or without preoperative *S*. *aureus* screening and decolonization after primary THA or TKA were complete; (3) sufficient data were provided for calculating pooled odds ratios (OR) with a 95% confidence interval (CI); (4) specific information on the measures of *S*. *aureus* screening and decolonization as well as the antibiotic application perioperatively was available.

The following exclusion criteria were used: (1) those studies that the data of interest were incomplete; (2)patients were treated with emergency joint replacements (e.g., hip arthroplasty for femoral neck fracture) or revision surgeries; (3) research contained other orthopedic surgeries; (4) full manuscript was not available; (5) reduplicative studies of the same patients in different periods; (6) reviews, commentaries, editorials, and letters were excluded; (7) languages were not accessible to authors.

### Study quality assessment

The methodological study quality was assessed using the Newcastle-Ottawa Scale (NOS), a validated tool suitable for cohort and case-control study [17]. Two reviewers assessed the studies independently, resolved divergences through discussions or reached consensus with the third author.

### Data extraction

Pertinent data were extracted by two reviewers independently from all eligible studies using a standardized data collection form, including the following variables: author, year of publication, country, type of study, number of TJR, type of arthroplasty (THA, TKA, or both), the methods of *S*. *aureus* screening and decolonization, application of perioperative antibiotics, definition of PJI, and any wound complications, number of SSI (PJI and superficial infection), and infection rates of *S*. *aureus* and other bacteria. For research lacking the results we needed, we had contacted the author(s) via email for more information.

The primary outcome of this research was SSI. However, different studies followed various definitions of SSI and PJI. Therefore, similar to Bedard et al. [7], “any wound complications” and “PJI” were used to pool end points of reported infection. “Any wound complications” was defined as any postoperative wound complications reported in the included studies, while PJI was defined as any deep infection involving prosthesis. The secondary outcome was the infection rate of *S*. *aureus* and other bacteria between the two groups.

### Statistical analysis

The OR and associated 95% CI were used to perform estimates for dichotomous variables, while the mean ± standard deviation was used for continuous variables. Only research that contained both OR and CI could be included in the analysis. *P* values < 0.05 were considered to be statistical significance.

The *I*2 and *p* value were used to evaluate the statistical heterogeneity across studies. If the heterogeneity test indicated the *p* values > .1 or *I*^2^ < 50%, the fixed effect model would be applied. On the contrary, if the heterogeneity test expressed the *p* values ≤ .1 or *I*^2^ ≥ 50%, the random effect model would be used. If necessary, a sensitivity analysis was conducted to assess the stability of the results. If data were available, subgroup analysis was also conducted to get more specific conclusions. In addition, forest plots were used to depict the results of each study and evaluate pooled estimates respectively, and the funnel plots were used to evaluate publication bias. All statistical analyses were performed through Review Manager (version 5.3.5 for Windows; The Cochrane Collaboration, The Nordic Cochrane Center, Copenhagen, 2014).

## Results

### Study selection

**Fig. 1:**
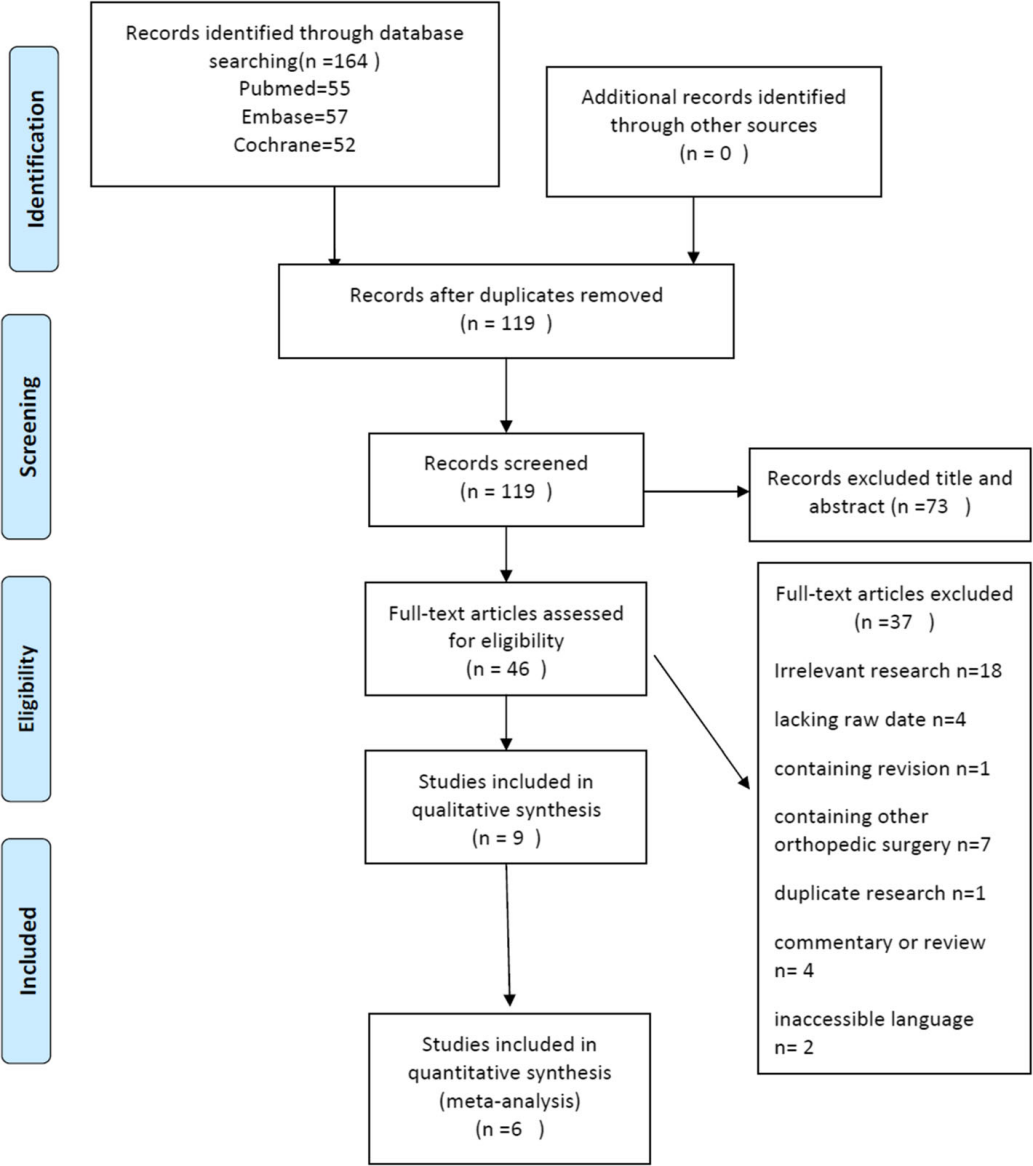
Summary of the evidence search and selection process

A total of 164 potentially relevant articles were identified from the three electronic databases through systematic search. Forty-five duplicates were deleted by citation management software and manual review of records. After two authors reviewed the titles and abstracts, 73 irrelevant citations were removed. The remaining 46 full-text papers were then retrieved for a more detailed analysis, of which 37 papers were excluded for several reasons, such as irrelevant research (*n* = 18), lacking raw date (*n* = 4) [18–21], containing revision or other orthopedic surgery (*n* = 8) [14, 22–28], duplicate research (*n* = 1) [29], commentary or review (*n* = 4) [20–33], and inaccessible language (*n* = 2) [34, 35]. Finally, a total of nine studies were included in this research [4, 10–13, 36–39]. Six of them were performed multivariate analysis [10–13, 37, 38], and the remaining three studies were given a descriptive analysis of findings because of slightly different intervention methods [4, 36, 39]. The study selection process throughout the different phases is shown in Fig. 1.

### Study characteristics and quality

A total of 36041 joint replacements were included in the nine studies, of which 26226 were divided into *S*. *aureus* screening and decolonization group, and the remaining 9815 were the control group. Except for one study that only included TKA [10], all other studies included TKA and THA [4, 11–13, 36–39]. Among the nine studies, six were from the USA [4, 11–13, 37, 38], two from the UK [36, 39], and one from Spain [10]. The longest follow-up period in these studies was 2 years [11], and the shortest follow-up time was 30 days [38], while it was difficult to find specific follow-up time in two studies [36, 39]. We tried to contact the authors, but failed. So we performed a statistical description of these two studies instead of the combination of effect sizes. Of the included studies, there were two prospective studies and seven retrospective studies with a score of 6 to 8 stars according to the NOS. Overall, these studies were of moderate to high quality. The detailed study qualities are shown in Appendix 2. A summary of the general characteristics, intervention measures, antibiotic prophylaxis, and outcomes of interests of all included studies in this review is displayed in Tables 1 and 2.

**Table 1:** Characteristics of the studies included in the review

Author	Year	Country	Study type	Number of TJA	Group	Age (years)	Gender F/M	BMI	Diagnosis	Type of TJA	Follow-Up	NOS
Stambough et al.	2017	USA	Retrospective	4186	Screening	THA 58.2 ± 13.5 TKA 63.1 ± 9.6	1146/903	THA 29.0 ± 5.0 TKA 32.1 ± 5.8	NM	1003TKA/1202THA	90 days	8
					Control	THA 57.2 ± 14.1 TKA 63.3 ± 10.0	1031/833	THA 29.3 ± 5.9 TKA 32.9 ± 6.4	NM	836TKA/1145THA		
Hofmann et al.	2017	USA	Retrospective	1034	Screening	61.5 ± 11.6	324/214	NM	degenerative joint disease	306TKA/232 THA	≥ 1 year	8
					Control	60.6 ± 12.3	279/217	NM		294TKA/202THA		
Hadley et al.	2010	USA	Retrospective	2058	Screening	NM	NM	NM	NM	TKA THA	1 year	7
					Control	NM	NM	NM	NM			
Sankar et al.	2005	UK	Prospective	395	Screening	68.53 (48–77)	141/90	NM	OA: 381, RA: 12, AS: 2	106TKA/125THA	NM	7
					Control	67.39 (51–79)	106/58	NM		79TKA/85THA		
Jeans et al.	2018	UK	retrospective	12911	Screening	68.6 (16–99)	5125/4193	29.9 (15.6–61)	NM	TKA THA	NM	6
					Control	68.5 (22–100)	1940/1653	29.5 (17.3–50.2)	NM			
Rao et al.	2011	USA	Prospective	2026	Screening	NM	NM	NM	NM	TKA THA	2 years	6
					Control	NM	NM	NM	NM			
Hacek et al.	2008	USA	Retrospective	1495	Screening	69.0	565/347	NM	NM	(709TKA/786THA)	1 year	8
					Control	69.1	356/227	NM	NM			
Pelfort et al.	2019	Spain	Retrospective	803	Screening	72.4 ± 6.9	278/125	NM	NM	TKA	1 year	8
					Control	72.2 ± 6.8	266/134	NM	NM			
Sporer et al.	2016	UKA	Retrospective	11133	Screening	NM	5819/3972	NM	NM	6175TKA/3661THA	30 days	7
					Control	NM	847/593	NM	NM	924TKA/ 516THA		

*TJA* total joint arthroplasty, *F* female, *M* male, *BMI* body mass index, *THA* total hip arthroplasty, *TKA* total knee arthroplasty, *NM* not mentioned, *NOS* Newcastle-Ottawa Scale, *OA* osteoarthritis, *RA* rheumatoid arthritis, *AS* ankylosing spondylitis

**Table 2:** Summary of intervention measures, antibiotic prophylaxis and outcomes of interests for each study

Author	Screening method (who receive the inteventions)	inteventional measures (number of days)	Antibiotic prophylaxis	Definition of PJI	Definition of “Any Wound complications”	Intervention group		Control group	
						Events (PJI/superficial)	Total	Events (PJI/superficial)	Total
Stambough et al. 2017	No screen (all patients)	Mupirocin intranasally 2 daily and chlorhexidine showers 5 days	CAP. High-risk groups and MRSA carriers were given vancomycin in addition to weight-based cefazolin.	MSIS	NHSN guidelines	5	2205	15	1981
Hofmann et. al. 2017	No screen (all patients)	Nasal application of mupirocin 2 daily for 5 days	CAP. the intervention group received 1 g of vancomycin as a single preoperative dose.	CDC	NHSN and CDC	4(1/3)	538	10(7/3)	496
Hadley et al. 2010	Nasal swab cultured (all patients)	Mupirocin intranasally and chlorhexidine showers 5 days	CAP. MRSA carriers were given Vancomycin 1 g at least 30 min before incision and every 12 h lasting for 24 h.	NM	CDC. Only deep incisional SSIs were considered clinically relevant and considered in the analysis.	21 (21/0)	1644	6 (6/0)	414
Sankar et al. 2005	Axilla, nose, groin, and any open wounds swab (positive screening results)	Mupirocin or povidone iodine or triclosan was instituted.	cephAzolin or cefuroxime was given one hour before skin incision and continued for 2 more doses 8 and 16 h post-operatively.	NM	NM	0	231	1	164
Jeans et al. 2018	Nasal and groin swabs culture (positive screening results)	Bactroban intranasally 4 times/day and bathe with Octenisan for 5 days.	NM	Public Health England’s published standard	Public Health England’s published standard on superficial and deep infection.	131	9318	69	3593
Rao et al. 2011	Nasal swab cultures (positive screening results)	Mupirocin intranasally 2 times daily and bathe with chlorhexidine daily for 5 days	CAP. Patients with a history of MRSA infection or for MRSA carriers received vancomycin 1 g 60 min before surgery followed by 1 g every 12 hours for 24 h.	NM	NM	17 (8/9)	1285	20 (9/11)	741
Hacek et al. 2008	Nasal swab real-time PCR (positive screening results)	Mupirocin 2 daily for 5 days	Hip surgery patients received cefazolin prior to surgery while knee surgery patients received vancomycin.	CDC	CDC	11(2/8)	912	14(0/14)	583
Pelfort et al. 2019	Nasal swab culture (positive screening results)	Mupirocin intranasally 3 times daily and chlorhexidine showers 5 days	CAP. MRSA carriers received 1 g of vancomycin.	MSIS	CDC. The superficial infections were confirmed by a positive culture of the drainage of the surgical incision together with the local symptoms and a negative intraarticular culture.	5 (3/2)	403	17 (10/7)	400
Sporer et al. 2016	Nasal swab culture (positive screening results)	Mupirocin intranasally 2 daily along with chlorhexidine showers 5 days	CAP patients who tested positive for MRSA were treated with vancomycin within 2 h preoperatively.	NM	Criteria for SSI included purulent drainage from the wound, serosanguinous drainage from an erythematous incision with a positive wound culture or a note in the medical record.	33	9690	16	1443

*CAP* conventional antibiotic prophylaxis (patients received a weight-based dose of cefazolin within 1 h before surgical incision and continued for 24 h postoperatively), *MSIS* Musculoskeletal Infection Society criteria, *NHSN* National Healthcare Safety Network (a deep SSI was defined as an infection having involvement below fascia, or having one of the following intraoperative findings: deep purulence surrounding the capsule or wound and fascial dehiscence with a positive deep tissue culture or secondary signs of infection), *SSIs* surgical site infections, *CDC* Center for Disease Control (The type of infection was recorded as superficial incisional, deep incisional, or organ space/joint)

### Strategies of antibiotic prophylaxis

Different studies had various strategies for antibiotic prophylaxis. The most common methods of antibiotic prophylaxis were as follows: patients received a weight-based dose of cefazolin within 30–60 min before surgical incision and continued for 24 h postoperatively in both groups, while methicillin-resistant *S*. *aureus* (MRSA) carriers were treated with vancomycin 1 g at least 30 min before incision and every 12 h lasting for 24 h in screening group. However, in the study performed by Hofmann et al. [37], all patients received 1 g of vancomycin preoperative in the screening group. In another study of Hacek et al. [13], hip surgery patients were treated with cefazolin preoperatively while knee surgery patients were given vancomycin in the screening group.

### Definition of SSI

The majority of studies included in this review used the diagnostic criteria of Center for Disease Control (CDC) and/or National Healthcare Safety Network (NHSN) for any wound complications [4, 10, 12, 13, 37], while Jeans et al. [36] used the Public Health England’s published standard, Sporer et al. [38] diagnosed SSI based on clinical symptoms, positive wound culture, and a note in the medical record, while Rao et al. [11] and Sankar et al. [39] did not give a specific definition.

### *S*. *aureus* decolonization and SSIs

**Fig. 2:**
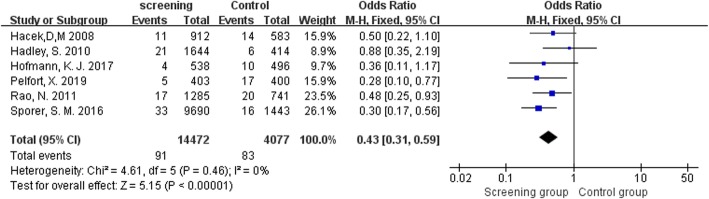
Comparison of SSIs between Screening group and control group

**Fig. 3:**
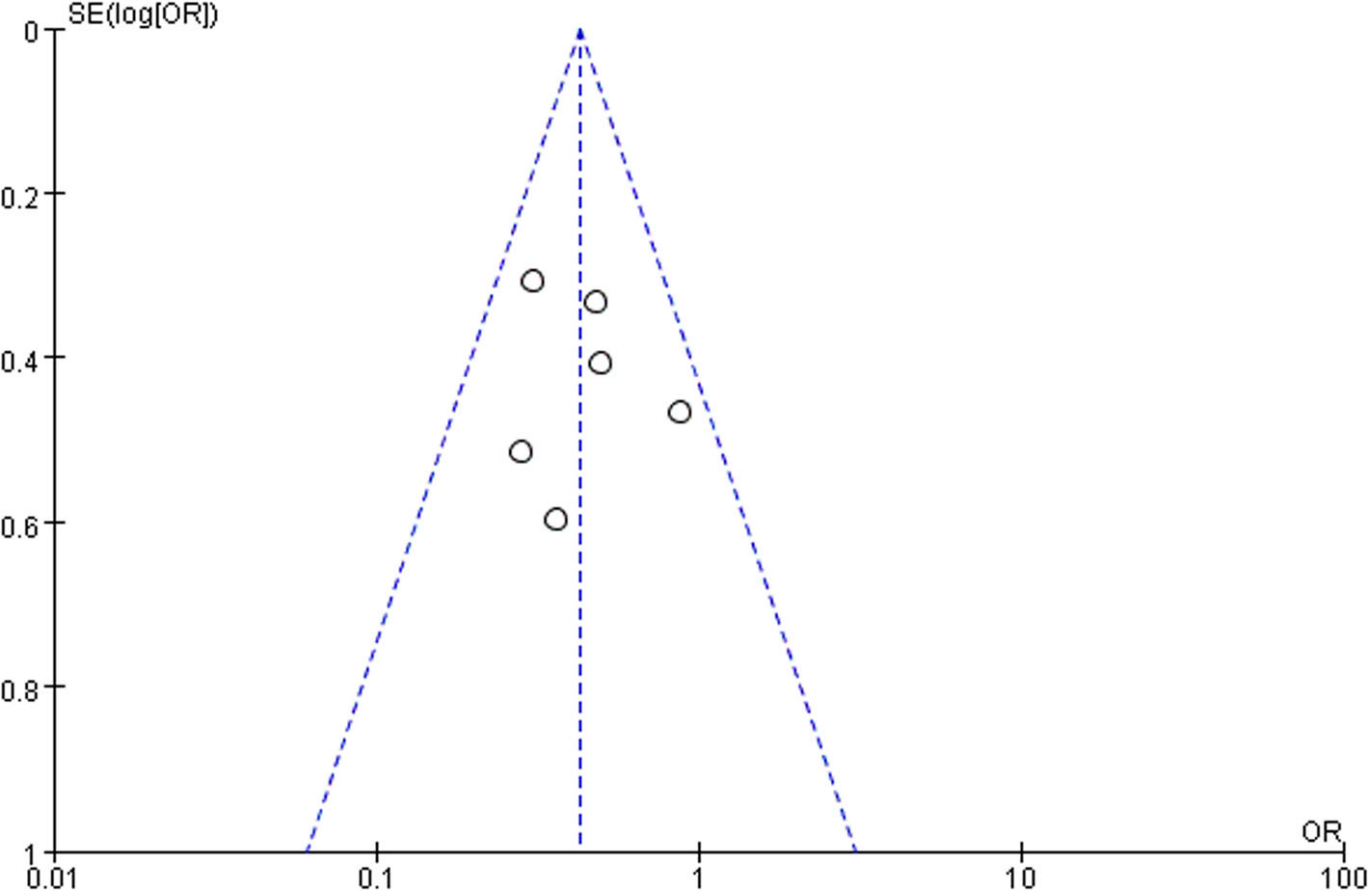
Funnel plot illustrating a meta-analysis of the SSIs. *SE* standard error, *OR* odds ratio

Six studies [10–13, 36–38] that assessed 18549 TJA reported postoperative SSI. The pooled analyses demonstrated that *S*. *aureus* screening and decolonization prior to surgery decreased the risk of subsequent SSI (OR = 0.43, 95% CI 0.31 to 0.59, *I*^2^ = 0%, *p* < 0.001; Fig. 2). The funnel plots indicated no obvious publication bias (Fig. 3).

Due to varying intervention methods used in the original studies, three studies could not be included in pooled data [4, 36, 39]. Stambough et al. [4] found that both the overall SSI rate (5 vs. 15 cases, *p* = .013) and SSI caused by *S*. *aureus* (2 vs. 10, *p* = .01) were significantly decreased; however, compared with traditional methods, the patients in the screening group were all given vancomycin prior to surgery, and all patients in the control group were also screened for *S*. *aureus* colonization and selectively treated with mupirocin preoperatively. Sankara et al. [39] found that there was a significant reduction in hospital-acquired infection rate following the screening for MRSA prior to TJR. Jeans et al. [36] also demonstrated that screening and eradication of methicillin-sensitive *S*. *aureus* (MSSA) could not only effectively reduce the incidence of MSSA PJI but also save a lot of costs. Nevertheless, except for nasal screening of *S*. *aureus* colonization, these two studies also screened groin and/or armpit [36, 39].

**Fig. 4:**
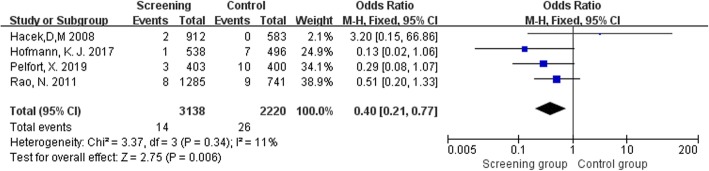
Comparison of PJI between screening group and control group

**Fig. 5:**
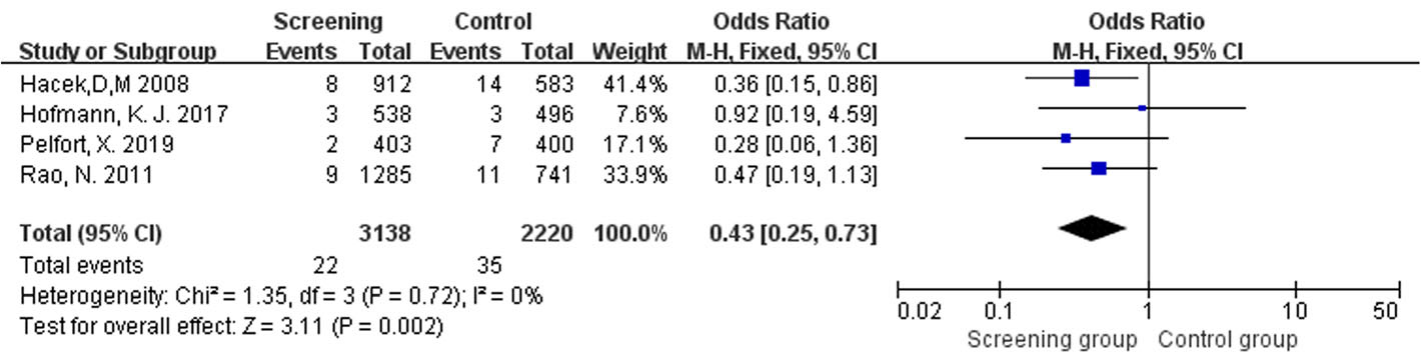
Comparison of superficial infection between Screening group and control group

Only four studies provided relevant information about deep and superficial infection [10, 11, 13, 37]. Infection category was not distinguished in the study by Sporer et al. [38], while only deep infection was included in the analysis of Hadley et al. [12]. To assess whether there was a difference in the effects of *S*. *aureus* screening and decolonization on PJI and superficial infection, we performed a subgroup analysis. The pooled analyses demonstrated significant statistical differences between the two groups both in PJI (OR = 0.40, 95% CI 0.21 to 0.77, *I*^2^ = 11%, *p* = 0.006; Fig. 4) and superficial infection (OR = 0.43, 95% CI 0.251 to 0.73, *I*^2^ = 0%, *p* = 0.002; Fig. 5).

**Fig. 6:**
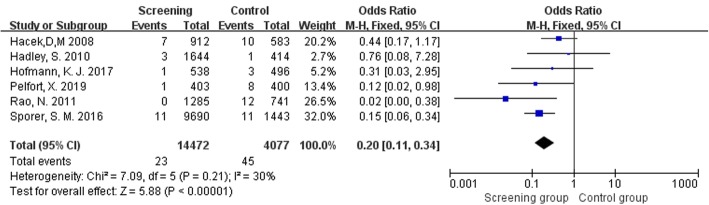
Comparison of SSIs caused by *S*. *aureus* between screening group and control group

**Fig. 7:**
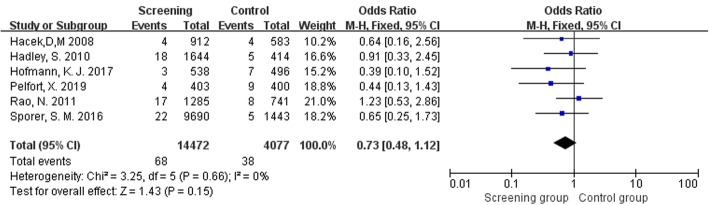
Comparison of SSIs caused by other bacteria between screening group and control group

Six studies [10–13, 37, 38] including 18549 TJR assessed postoperative SSI caused by *S*. *aureus* or other bacteria. Table 3 presents detailed information on the distribution of *S*. *aureus* and other bacteria in SSI with or without *S*. *aureus* screening and decolonization. The pooled data showed a significant statistical difference in postoperative *S*. *aureus* infection between the two groups (OR = 0.20, 95% CI 0.11 to 0.34, *I*^2^ = 30%, *p* < 0.001; Fig. 6). However, there was no statistical difference in SSI caused by other bacteria (OR = 0.73, 95% CI 0.48 to 1.12, *I*^2^ = 0%, *p* = 0.15; Fig. 7).

**Table 3:** Distribution of *S*. *aureus* and other bacteria in SSIs

Author	Screening group		Control group	
	*S*. aureus/other bacterial infection	Total	*S*. *aureus*/other bacterial infection	Total
Hofmann et al. 2017	1/3	538	3/7	496
Hadley et al. 2010	3/18	1644	1/5	414
Rao et al. 2011	0/17	1285	12/8	741
Hacek et al. 2008	7/4	912	10/4	583
Pelfort et al. 2019	1/4	403	8/9	400
Sporer et al. 2016	11/22	9690	11/5	1443

## Discussion

This systematic review and meta-analysis included nine independent studies that analyzed 36041 cases of arthroplasty and directly assessed the effectiveness of *S*. *aureus* decolonization and nondecolonization in SSI following primary THA and TKA procedures. The pooled analyses indicated that *S*. *aureus* screening and decolonization reduced the SSI (both PJI and superficial infection). And further research suggested a decrease in SSI caused by *S*. *aureus*, while there was no difference in SSI caused by other bacteria between the two groups.

The nasal cavity is one of the most common sites for *S*. *aureus* colonization. One study revealed that the nasal colonization rate of *S*. *aureus* was 22.2% and that of MRSA was 0.8% [15]. Another study also showed that colonization rate of MSSA was 22.6% and that of MSRA was 4.4% [40]. It is generally believed that *S*. *aureus* in the nasal cavity is one of the potential sources of bacterial seeding for SSI. The methods of *S*. *aureus* screening mainly include bacterial culture and real-time polymerase chain reaction (RT-PCR).

Currently, the common method for *S*. *aureus* decolonization is that topical application of mupirocin twice daily to both nares accompany with or without chlorhexidine showers or skin wipes daily for 5 days prior to the TJA [41]. However, several studies implemented an extensive decolonization program, regardless of whether *S*. *aureus* was positive or not [4, 12, 37], which may be considered an abuse of antibiotics and may lead to an increased risk of bacterial resistance. Therefore, it may be more reasonable to perform selective decolonization programs for only screened positive patients.

Conventional application of this regimen to patients with positive *S*. *aureus* preoperative has shown good short-term results in improving decolonization rates [4]. However, its long-term efficacy is uncertain. Many studies showed that a significant number of patients would remain positive for *S*. *aureus* after surgery [42, 43]. For example, a study revealed that 33% (19 of 58) of patients still performed positive for *S*. *aureus* colonization at 3–30 months after surgery despite preoperative decolonization [44]. Another recent study also showed that the screening and decolonization regimen did significantly reduce the number of nasal carriage of MRSA, but the study also showed that about 20% of patients might remain colonized by MRSA despite a decolonization protocol in patients undergoing Elective TJA [28]. This means that a decolonization treatment could not guarantee that patients will keep decolonized in the future, which requires further research, because it is still unclear whether the risk of late infection in this population is higher [44].

So far, whether the *S*. *aureus* decolonization program could reduce the SSI in patients undergoing primary TJA is still controversial. Numerous studies showed that SSI could be decreased by an institutional nasal screening and decolonization protocol for patients before elective TJA regardless of carriers of either MSSA or MRSA [38, 40]. For instance, Sporer et al. [38] demonstrated a 69% reduction in the prevalence of SSI after the use of screening and decolonization program. Similar to this study, Kim et al. [40] also reported a 59% reduction in SSI after implementing a similar screening protocol, and they found that MRSA carriers had a higher rate of SSI than that of non-carriers (0.97% vs. 0.19%). Therefore, nasal carriage of *S*. *aureus* has been considered as an independent risk factor for SSI and PJI. Conversely, several recent studies demonstrated that nasal decolonization protocol prior to elective TJA could not decrease the incidence of SSI [14, 15, 28]. After comparing the infection rates of colonized and noncolonized patients, Ramos et al. [14] discovered that the risk of infection in decolonized patients could not be decreased to the baseline level of nondecolonized patients. A prospective randomized trial also demonstrated that there was no significant difference between treated and untreated carriers and that most of *S*. *aureus* PJI occurred in non-carriers, so the authors believed that there was no clear benefit in screening and/or decolonizing carriers before TJA [15].

Based on the included studies, our pooled data showed that *S*. *aureus* screening and decolonization dramatically reduced the incidence of SSI. However, it should be noted that in these studies, patients screened positive for MRSA also received vancomycin as a standard perioperative antibiotic prophylaxis, so it did not rule out that the decrease of infection rate might be caused by the use of vancomycin, which needs to be further studied. Additionally, Jeans et al. [36] reported that MSSA screening and decolonization might be more effective in decreasing overall infection rate in hips than in knees. Unfortunately, due to insufficient data in this area, we are unable to conduct relevant analysis.

To the best of our knowledge, this is the most comprehensive meta-analysis examining the association of *S. aureus* screening and decolonization with SSI after TKA and THA. We are aware of previous meta-analyses on this topic [30, 45, 46]. Studies performed by Levy et al. [45] and Chen et al. [46] contained other orthopedic surgeries, which would lead to a mass of confounding factors. In another very recent paper performed by Sadigursky et al. [30], only four studies were included in the analysis. Compared with these studies, we included some new clinical studies up to 2019. Moreover, we strictly assessed the quality of all selected studies and followed the PRISMA statement in this study.

Although we have designed and implemented the research as perfectly as possible, our meta-analysis still inevitably has several inherent limitations. First, as all systematic reviews, some studies would be ignored because of search strategies. In order to overcome this problem as much as possible, we consulted professional librarians and optimized search strategies continuously during the search process. Second, most of the included studies were retrospective rather than prospective, and follow-up duration of some selected studies was short (about 30–90 days), which might prevent late PJI and SSI from being observed. Third, the diagnostic criteria of PJI and SSI in different studies were not uniform, and preoperative treatment with vancomycin might affect the outcomes for patients screened positive for MRSA. Finally, the data analysis in this paper were performed at the research level rather than at the patient level; therefore, we could not take each patient's physical status, age, gender, diagnoses, body mass index, American society of anesthesiologist (ASA), duration of surgery, nutritional status, and follow-up times into account, all of which might affect the results. Given the above shortcomings, more prospective and long-term follow-up studies are still needed to better understand the impact of the *S. aureus* screening and decolonization on the incidence of SSI and PJI after TJA.

## Conclusions

In conclusion, the results of this systematic review and meta-analysis suggested that *S. aureus* screening and decolonization prior to elective primary THA and TKA significantly decreased the risk of SSI and PJI. Therefore, it was our recommendation to implement a standardized universal screening and selective decolonization regimen for all patients undergoing elective TKA and THA. However, these findings were based upon retrospective studies, so lager-scale prospective multicenter studies are needed to evaluate the impact of S. aureus screening and decolonization on SSI and PJI after TJA.

## Supplementary information

**Additional file 1.** Search Strategy.

**Additional file 2.** Risk-of-bias assessment for the studies included in the meta-analysis.

## Data Availability

The authors declare that all the data supporting the findings of this study are available within the article and its supplementary information files.

## Abbreviations

MRSA: Methicillin-resistant *S*. *aureus*
MSSA: Methicillin-sensitive *S*. *aureus*
PJI: Periprosthetic joint infection
*S*. *aureus*: Staphylococcus aureus
SSI: Surgical site infection
THA: Total hip arthroplasty
TJA: Total joint arthroplasty
TKA: Total knee arthroplasty
