# Supplementary material for: Can nasal Staphylococcus aureus screening and decolonization prior to elective total joint arthroplasty reduce surgical site and prosthesis-related infections? A systematic review and meta-analysis
Source: J Orthop Surg Res. 2020 Feb 19;15:60. doi: 10.1186/s13018-020-01601-0 (PMC7031963; doi:10.1186/s13018-020-01601-0)
Supplement: Supplementary file 1 — Additional file 1. Search Strategy. [file 13018_2020_1601_MOESM1_ESM.docx]

**Pubmed**

**#1**

"Arthroplasty, Replacement"[Mesh] OR Arthroplasty, Replacement[Title/Abstract] OR Arthroplasties, Replacement[Title/Abstract] OR Joint Prosthesis Implantation[Title/Abstract] OR Implantation, Joint Prosthesis[Title/Abstract] OR Implantations, Joint Prosthesis[Title/Abstract] OR Joint Prosthesis Implantations[Title/Abstract] OR Prosthesis Implantations, Joint[Title/Abstract] OR Prosthesis Implantation, Joint[Title/Abstract] OR Replacement Arthroplasty[Title/Abstract] OR Joint Replacement[Title/Abstract] OR Joint Replacements[Title/Abstract] OR Replacement, Joint[Title/Abstract] OR Replacements, Joint[Title/Abstract] OR Replacement Arthroplasties[Title/Abstract] OR Total Joint Replacement[Title/Abstract] OR Joint Replacement, Total[Title/Abstract] OR Joint Replacements, Total[Title/Abstract] OR Replacement, Total Joint[Title/Abstract] OR Replacements, Total Joint[Title/Abstract] OR Total Joint Replacements[Title/Abstract] OR Hip replacement[Title/Abstract] OR Hip replacements[Title/Abstract] OR Hip arthroplasty[Title/Abstract] OR Hip arthroplastic[Title/Abstract] OR Hip arthroplasties[Title/Abstract] OR Hip prosthesis[Title/Abstract] OR Hip reconstruction[Title/Abstract] OR Knee replacement[Title/Abstract] OR Knee replacements[Title/Abstract] OR Knee arthroplasty[Title/Abstract] OR Knee arthroplastic[Title/Abstract] OR Knee arthroplasties[Title/Abstract] OR Knee prosthesis[Title/Abstract] OR Knee reconstruction[Title/Abstract] OR TJR[Title/Abstract] OR TJA[Title/Abstract] OR TKR[Title/Abstract] OR TKA[Title/Abstract] OR THA[Title/Abstract] OR THR[Title/Abstract]

**#2**

“Staphylococcus aureus”[Mesh] OR “Staphylococcus”[Mesh] OR Staphylococcus aureus[Title/Abstract] OR Staphylococcus[Title/Abstract] OR Staphylococcal[Title/Abstract] OR S. aureus[Title/Abstract] OR MRSA[Title/Abstract] OR MSSA[Title/Abstract]

**#3**

“Administration, Intranasal” [Mesh] OR Administration, Intranasal[Title/Abstract] OR “nose” [Mesh] OR nose[Title/Abstract] OR intranasal[Title/Abstract] OR nasal[Title/Abstract]

**#4: #2 AND #3**

**#5**

“Prosthesis-Related Infections” [Mesh] OR Prosthesis-Related Infections[Title/Abstract] OR Prosthesis Related Infections[Title/Abstract] OR Infections, Prosthesis-Related[Title/Abstract] OR Prosthesis-Related Infection[Title/Abstract] OR “Surgical Wound Infection” [Mesh] OR Surgical Wound Infection[Title/Abstract] OR Infections, Surgical Wound[Title/Abstract] OR Surgical Wound Infections[Title/Abstract] OR Wound Infections, Surgical[Title/Abstract] OR Infection, Surgical Wound[Title/Abstract] OR Surgical Site Infection[Title/Abstract] OR Infection, Surgical Site[Title/Abstract] OR Infections, Surgical Site[Title/Abstract] OR Surgical Site Infections[Title/Abstract] OR Wound Infection, Postoperative[Title/Abstract] OR Wound Infection, Surgical[Title/Abstract] OR Infection, Postoperative Wound[Title/Abstract] OR Infections, Postoperative Wound[Title/Abstract] OR Postoperative Wound Infections[Title/Abstract] OR Wound Infections, Postoperative[Title/Abstract] OR Postoperative Wound Infection[Title/Abstract] OR “Postoperative Complications” [Mesh] OR Postoperative Complications[Title/Abstract] OR Complication, Postoperative[Title/Abstract] OR Complications, Postoperative[Title/Abstract] OR Postoperative Complication[Title/Abstract] OR PJI[Title/Abstract]

**#1 AND #4 AND #5**

**Embase**

**#1** ‘replacement arthroplasty’/exp

**#2**

‘Arthroplasty, Replacement’:ab,ti OR ‘Arthroplasties, Replacement’:ab,ti OR ‘Joint Prosthesis Implantation’:ab,ti OR ‘Implantation, Joint Prosthesis’:ab,ti OR ‘Implantations, Joint Prosthesis’:ab,ti OR ‘Joint Prosthesis Implantations’:ab,ti OR ‘Prosthesis Implantation, Joint’:ab,ti OR ‘Prosthesis Implantations, Joint’:ab,ti OR ‘Replacement Arthroplasty’:ab,ti OR ‘Joint Replacement’:ab,ti OR ‘Joint Replacements’:ab,ti OR ‘Replacement, Joint’:ab,ti OR ‘Replacements, Joint’:ab,ti OR ‘Replacement Arthroplasties’:ab,ti OR ‘Total Joint Replacement’:ab,ti OR ‘Joint Replacement, Total’:ab,ti OR ‘Joint Replacements, Total’:ab,ti OR ‘Replacement, Total Joint’:ab,ti OR ‘Replacements, Total Joint’:ab,ti OR ‘Total Joint Replacements’:ab,ti OR ‘Hip replacement’:ab,ti OR ‘Hip replacements’:ab,ti OR ‘Hip arthroplasty’:ab,ti OR ‘Hip arthroplastic’:ab,ti OR ‘Hip arthroplasties’:ab,ti OR ‘Hip prosthesis’:ab,ti OR ‘Hip reconstruction’:ab,ti OR ‘Knee replacement’:ab,ti OR ‘Knee replacements’:ab,ti OR ‘Knee arthroplasty’:ab,ti OR ‘Knee arthroplastic’:ab,ti OR ‘Knee arthroplasties’:ab,ti OR ‘Knee prosthesis’:ab,ti OR ‘Knee reconstruction’:ab,ti OR ‘TJR’:ab,ti OR ‘TJA’:ab,ti OR ‘TKR’:ab,ti OR ‘TKA’:ab,ti OR ‘THA’:ab,ti OR ‘THR’:ab,ti OR ‘acetabuloplasty’:ab,ti

**#3: #1 OR #2**

**#4** ‘Staphylococcus aureus’ /exp

**#5**

‘Staphylococcus aureus’:ab,ti OR ‘Staphylococcus’:ab,ti OR ‘Staphylococcal’:ab,ti OR ‘S. aureus’:ab,ti OR ‘MRSA’:ab,ti OR ‘MSSA’:ab,ti

**#6: #4 OR #5**

**#7**  ‘intranasal drug administration’/exp

**#8** ‘nose’ /exp

**#9**

‘intranasal drug administration’:ab,ti OR ‘nose’:ab,ti OR ‘intranasal’:ab,ti OR ‘nasal’:ab,ti

**#10: #7 OR #8 OR #9**

**#11: #6 AND 10**

**#12 'application site infection'/exp**

**#13**

‘Application Site Infection’:ab,ti OR ‘Prosthesis-Related Infections’:ab,ti OR ‘Prosthesis Related Infections’:ab,ti OR ‘Infections, Prosthesis-Related’:ab,ti OR ‘Prosthesis-Related Infection’:ab,ti OR ‘PJI’:ab,ti OR 'surgical infection'/exp OR ‘Surgical Infection’:ab,ti OR ‘Surgical Wound Infection’:ab,ti OR ‘Infections, Surgical Wound’:ab,ti OR ‘Surgical Wound Infections’:ab,ti OR ‘Wound Infections, Surgical’:ab,ti OR ‘Infection, Surgical Wound’:ab,ti OR ‘Surgical Site Infection’:ab,ti OR ‘Infection, Surgical Site’:ab,ti OR ‘Infections, Surgical Site’:ab,ti OR ‘Surgical Site Infections’:ab,ti OR ‘Wound Infection, Postoperative’:ab,ti OR ‘Wound Infection, Surgical’:ab,ti OR ‘Infection, Postoperative Wound’:ab,ti OR ‘Infections, Postoperative Wound’:ab,ti OR ‘Postoperative Wound Infections’:ab,ti OR ‘Wound Infections, Postoperative’:ab,ti OR ‘Postoperative Wound Infection’:ab,ti

**#14 'postoperative complication'/exp**

**#15 'infectious complication'/exp**

**#16**

‘Infectious Complication’:ab,ti OR ‘Postoperative Complications’:ab,ti OR ‘Complication, Postoperative’:ab,ti OR ‘Complications, Postoperative’:ab,ti OR ‘Postoperative Complication’:ab,ti

**#17**: **#12 OR #13 OR #14 OR #15 OR #16**

**#18**: **#3 AND #11 AND #17**

**Cochrane**

**#1**

MeSH descriptor: [Arthroplasty, Replacement] explode all trees

**#2**

(Arthroplasty, Replacement):ti,ab,kw OR (Arthroplasties, Replacement):ti,ab,kw OR (Joint Prosthesis Implantation):ti,ab,kw OR (Implantation, Joint Prosthesis):ti,ab,kw OR (Implantations, Joint Prosthesis):ti,ab,kw OR (Joint Prosthesis Implantations):ti,ab,kw OR (Prosthesis Implantation, Joint):ti,ab,kw OR (Prosthesis Implantations, Joint):ti,ab,kw OR (Replacement Arthroplasty):ti,ab,kw OR (Joint Replacement):ti,ab,kw OR (Joint Replacements):ti,ab,kw OR (Replacement, Joint):ti,ab,kw OR (Replacements, Joint):ti,ab,kw OR (Replacement Arthroplasties):ti,ab,kw OR (Total Joint Replacement):ti,ab,kw OR (Joint Replacement, Total):ti,ab,kw OR (Joint Replacements, Total):ti,ab,kw OR (Replacement, Total Joint):ti,ab,kw OR (Replacements, Total Joint):ti,ab,kw OR (Total Joint Replacements):ti,ab,kw OR (Hip replacement):ti,ab,kw OR (Hip replacements):ti,ab,kw OR (Hip arthroplasty):ti,ab,kw OR (Hip arthroplastic):ti,ab,kw OR (Hip arthroplasties):ti,ab,kw OR (Hip prosthesis):ti,ab,kw OR (Hip reconstruction):ti,ab,kw OR (Knee replacement):ti,ab,kw OR (Knee replacements):ti,ab,kw OR (Knee arthroplasty):ti,ab,kw OR (Knee arthroplastic):ti,ab,kw OR (Knee arthroplasties):ti,ab,kw OR (Knee prosthesis):ti,ab,kw OR (Knee reconstruction):ti,ab,kw OR (TJR):ti,ab,kw OR (TJA):ti,ab,kw OR (TKR):ti,ab,kw OR (TKA):ti,ab,kw OR (THA):ti,ab,kw OR (THR):ti,ab,kw OR (acetabuloplasty):ti,ab,kw

**#3: #1 OR #2**

**#4**

MeSH descriptor: [Staphylococcus aureus] explode all trees

**#5**

(Staphylococcus aureus):ti,ab,kw OR (Staphylococcus):ti,ab,kw OR (Staphylococcal):ti,ab,kw OR (S. aureus):ti,ab,kw OR (MRSA):ti,ab,kw OR (MSSA):ti,ab,kw

**#6: #4 OR #5**

**#7**

MeSH descriptor: [Administration, Intranasal] explode all trees

**#8**

MeSH descriptor: [Nose] explode all trees

**#9**

(Administration, Intranasal):ti,ab,kw OR (nose):ti,ab,kw OR (intranasal):ti,ab,kw OR (nasal):ti,ab,kw

**#10: #7 OR #8 OR #9**

**#11: #6 AND 10**

**#12 MeSH descriptor: [Prosthesis-Related Infections] explode all trees**

**#13 MeSH descriptor: [Postoperative Complications] explode all trees**

**#14 MeSH descriptor: [Surgical Wound Infection] explode all trees**

**#15**

(Prosthesis-Related Infections):ti,ab,kw OR (Prosthesis Related Infections):ti,ab,kw OR (Infections, Prosthesis-Related):ti,ab,kw OR (Prosthesis-Related Infection):ti,ab,kw OR (Surgical Wound Infection):ti,ab,kw OR (Infections, Surgical Wound):ti,ab,kw OR (Surgical Wound Infections):ti,ab,kw OR (Wound Infections, Surgical):ti,ab,kw OR (Infection, Surgical Wound):ti,ab,kw OR (Surgical Site Infection):ti,ab,kw OR (Infection, Surgical Site):ti,ab,kw OR (Infections, Surgical Site):ti,ab,kw OR (Surgical Site Infections):ti,ab,kw OR (Wound Infection, Postoperative):ti,ab,kw OR (Wound Infection, Surgical):ti,ab,kw OR (Infection, Postoperative Wound):ti,ab,kw OR (Infections, Postoperative Wound):ti,ab,kw OR (Postoperative Wound Infections):ti,ab,kw OR (Wound Infections, Postoperative):ti,ab,kw OR (Postoperative Wound Infection):ti,ab,kw OR (Postoperative Complications):ti,ab,kw OR (Complication, Postoperative):ti,ab,kw OR (Complications, Postoperative):ti,ab,kw OR (Postoperative Complication):ti,ab,kw OR (PJI):ti,ab,kw

**#16: #12 OR #13 OR #14 OR #15**

**#17: #3 AND #11 AND #16**
