# Supplementary material for: Can nasal Staphylococcus aureus screening and decolonization prior to elective total joint arthroplasty reduce surgical site and prosthesis-related infections? A systematic review and meta-analysis
Source: J Orthop Surg Res. 2020 Feb 19;15:60. doi: 10.1186/s13018-020-01601-0 (PMC7031963; doi:10.1186/s13018-020-01601-0)
Supplement: Supplementary file 2 — Additional file 2. Risk-of-bias assessment for the studies included in the meta-analysis. [file 13018_2020_1601_MOESM2_ESM.docx]

**Appendix 2** Risk-of-bias assessment for the studies included in the meta-analysis

| Study | Item 1 | Item 2 | Item 3 | Item 4 | Item 5A | Item 5B | Item 6 | Item 7 | Item 8 | Score |
| --- | --- | --- | --- | --- | --- | --- | --- | --- | --- | --- |
| Stambough et al | * | * | * | * | * | * | * |  | * | 8 |
| Hofmann et. al | * | * | * | * | * | * | * |  | * | 8 |
| Hadley et al | * | * | * | * | * | * |  |  | * | 7 |
| Sankar et al | * | * | * | * | * |  | * |  | * | 7 |
| Jeans et al | * | * | * | * |  |  | * |  | * | 6 |
| Rao et al | * | * | * | * |  |  | * |  | * | 6 |
| Hacek et al | * | * | * | * | * | * | * |  | * | 8 |
| Pelfort et al | * | * | * | * | * | * | * |  | * | 8 |
| Sporer et al | * | * | * | * |  | * | * |  | * | 7 |

Items in cohort studies: 1, representativeness of exposed cohort; 2, selection of the non-exposed cohort; 3, ascertainment of exposure; 4, demonstration that outcome of interest was not present at start of study; 5A, comparability of cohorts (on the basis of the design or analysis) regarding age; 5B, comparability of cohorts regarding disease extent and disease duration; 6, assessment of outcome; 7, follow-up was long enough for outcomes to occur; 8, adequacy of follow-up of cohorts.
